# Antibacterial activity and antibacterial mechanism of flavaspidic acid BB against *Staphylococcus haemelyticus*

**DOI:** 10.1186/s12866-023-02997-5

**Published:** 2023-09-29

**Authors:** Jiaxin Liu, Ruijie Liu, Rongrong Deng, Shiqian Zheng, Zhibin Shen

**Affiliations:** 1 School of Traditional Chinese Medicine, Guangdong Pharmaceutical University, Guangzhou, Guangdong Province China; 2 Guangdong Provincial Engineering Center of Topical Precise Drug Delivery System, Guangdong Pharmaceutical University, Guangzhou, Guangdong Province China; 3 Guangdong Cosmetics Engineering and Technology Research Center, Guangdong Pharmaceutical University, Guangzhou, Guangdong Province China

**Keywords:** *Dryopteris fragrans* (L.) Schott, Flavaspidic acid BB, *Staphylococcus haemolyticus*, Anti-bacterial activity, Anti-biofilm activity, Molecular docking

## Abstract

**Background:**

*Staphylococcus haemolyticus* (*S. haemolyticus*) is the main etiological factor in skin and soft tissue infections (SSTI). *S. haemolyticus* infections are an important concern worldwide, especially with the associated biofilms and drug resistance. Herein, we investigated the inhibitory effect of Flavaspidic acid BB obtained from plant extractions on clinical *S. haemolyticus* strains and their biofilms. Moreover, we predicted its ability to bind to the protein-binding site by molecular simulation. Since the combination of Hsp70 and RNase P synthase after molecular simulation with flavaspidic acid BB is relatively stable, enzyme-linked immunosorbent assay (ELISA) was used to investigate Hsp70 and RNase P synthase to verify the potential antimicrobial targets of flavaspidic acid BB.

**Results:**

The minimum inhibitory concentrations (MIC) of flavaspidic acid BB on 16 clinical strains of *S. haemolyticus* was 5 ~ 480 µg/mL, and BB had a slightly higher inhibitory effect on the biofilm than MUP. The inhibitory effect of flavaspidic acid BB on biofilm formation was better with an increase in the concentration of BB. Molecular simulation verified its ability to bind to the protein-binding site. The combination of ELISA kits showed that flavaspidic acid BB promoted the activity of Hsp70 and inhibited the activity of RNase P, revealing that flavaspidic acid BB could effectively inhibit the utilization and re-synthesis of protein and tRNA synthesis, thus inhibiting bacterial growth and biofilm formation to a certain extent.

**Conclusions:**

This study could potentially provide a new prospect for the development of flavaspidic acid BB as an antibacterial agent for resistant strains.

**Supplementary Information:**

The online version contains supplementary material available at 10.1186/s12866-023-02997-5.

## Background

Skin and soft tissue infections (SSTIs) are a common reason for patients seeking inpatient and outpatient medical care with more than 14 million outpatient visits a year [1]. According to the results of epidemiological statistical studies on SSTI, *Staphylococcus* is the most common pathogenic bacteria causing SSTI [2–4], and *S. haemolyticus* is one of the main pathogens related to SSTIs [5, 6]. With the widespread clinical application of broad-spectrum antimicrobials, drug resistance of opportunistic pathogenic bacteria associated with SSTI has become increasingly serious. Infections caused by coagulase-negative *Staphylococci* (CoNS) represented by *S. haemolyticus* are increasing every year [5–8]. In addition, *S. haemolyticus* may cause septicemia, peritonitis, otitis, and urinary tract infections [5].

Recently, bacterial infection in SSTI has attracted increasing attention in the academic field because of its high recurrence rate. The main reason for this is the ability of *Staphylococcus* to form biofilms that protect bacteria from host defenses and prevent the release of some antibiotics [7, 9, 10]. The biofilm state of bacteria is different from the planktonic state, which is a special developmental stage adopted by bacteria to adapt to the external environment. Bacterial biofilm development consists of four successive stages: (1) attachment, (2) aggregation, (3) maturation, and (4) dispersion [11]. Biofilm eradication requires 10-1000 times the minimum inhibitory concentration (MIC) of antibiotics normally needed to inhibit the planktonic form [12]. The literature indicates that *S. haemolyticus* can exhibit high levels of antimicrobial resistance and the ability to form strong biofilms [13]. Linical isolates of *S. haemolyticus* are more antibiotic resistant and have different versions of genes encoding their surface proteins [14]. *S. haemolyticus* isolates are more frequently multidrug resistant than other CoNS known to exhibit resistance to multiple anti-staphylococcal agents [5, 15]. In clinics, E, MUP, and FD are mainly used to treat SSTI infections. Previous studies have shown that these antibiotic agents have high rates of resistance to staphylococci [16, 17]. The remarkable characteristic of presenting high rates of antimicrobial resistance makes *S. haemolyticus* an emerging threat to human health. Therefore, finding a drug that can effectively inhibit the production of biofilm by bacteria and reduce the drug resistance of pathogens with a view to treating diseases caused by S. *haemolyticus* has become a hot topic in research.

With the rapid development of modern scientific research technology, molecular docking and molecular dynamics (MD) simulations have become important methods of studying the interaction between compound molecules and receptors [18, 19]. Molecular docking can predict the interaction between the receptor and the ligand. The stability of binding between the receptor and the ligand was studied through MD simulations at the molecular level. Both of them greatly save experimental materials and time for studying the mechanism of action of compounds and provide a basis for verification experiments. Owing to the abundance of antibacterial mechanisms and the early research of our group and to accurately and simply find the target quickly, we used molecular docking and MD simulation to predict potential targets and conducted enzyme dynamic experiments for verification.

*Dryopteris fragrans* (L.) *Schott* (*D. fragrans*), a member of the genus Dryopteris (*Dryopteris Adans.*), has been used for a variety of dermatological diseases such as psoriasis, acne, rash, and dermatitis. Different studies [20–23] have confirmed that phloroglucinol is the main active component of *D. fragrans*. Our group isolated a variety of phloroglucinols from the plant, and the experiment showed that the phloroglucinols, especially flavaspidic acid BB [24], isoflavaspidic acid PB [23], and aspidin BB [25], had strong antibacterial activity.

However, There are no studies on the effect of flavaspidic acid BB on biofilms of *S. haemolyticus*. Therefore, in this study, flavaspidic acid BB was used to increase its production and provide a number of raw materials for the study of its anti-bacterial and anti-biofilm activities against *S. haemolyticus*. Molecular docking and molecular dynamics verified its ability to bind to the protein-binding site. The antibacterial mechanism of flavaspidic acid BB on Hsp70 and RNase P synthase was investigated using enzyme-linked immunosorbent assay (ELISA). In this study, we have provided a simple and rapid method of predicting the potential targets of drugs. We screened out compounds with significant antibacterial activity against strains with strong clinical drug resistance and preliminarily explored their antibacterial mechanisms. Furthermore, This research provides experimental data and the theoretical basis for the development of flavaspidic acid BB as a new type of biofilm inhibitor and guidance for the clinical treatment of SSTI.

## Methods

### Antimicrobial agents and chemicals

Flavaspidic acid BB (BB, purity > 95%) was isolated from *D. fragrans* using silica gel column chromatography, Sephadex LH-20 column chromatography, and pre-HPLC with some modifications in the preliminary experiments [26–28]. Erythromycin (E, N0825A, 85%), mupirocin (MUP, 17,090,377, 95%), fusidic acid (FD, Z19J6Q1, 98%), cefazolin (190,501, 98%) and dimethyl sulfoxide (DMSO) were purchased from Dalian Meilun Biotechnology Inc. (Dalian, China), Sino-US Tianjin SK Pharmaceutical Inc. (Tianjin, China), Shanghai Yuanye Biotechnology Inc. (Shanghai, China), Guangdong South China Pharmaceutical Group Inc. (Guangzhou, China), and Sigma-Aldrich (Saint Louis, USA), respectively. Flavaspidic acid BB was dissolved in DMSO at a concentration of 51.2 mg/mL. E, MUP, and FD were dissolved in DMSO as a drug reserve solution (512 mg/ml) and stored at -20 °C.

### Bacterial strain and growth conditions

*S. aureus* (ATCC@29,213) quality control (QC) strain was purchased from the GuangDong Culture Collection Center. Sixteen clinical isolates of *S. haemolyticus* were obtained from Guangdong Lewwin Pharmaceutical Research Institute Co., Ltd. Bacteria were precultured on nutrient agar (NA) medium at 35 °C for 24 h, then incubated in Caton-adjusted Mueller-Hnton broth (CAMHB) or tryptone soy broth (TSB) medium and diluted to 1 × 10^6^ CFU/mL. All bacterial culture media were obtained from Sigma Aldrich (Saint Louis, USA).

### Determination of the MIC and MBC

The MIC (The minimum inhibitory concentrations) and MBC (the minimum bactericidal concentration) assays were performed by the microdilution method according to the Clinical and Laboratory Standards Institute [29–31]. In short, Add 1 × 10^6^ CFU/mL of the bacterial strain to individual wells of a microtiter plate after double dilution of test agent (2560 ~ 5 µg/mL of Flavaspidic acid BB and antimicrobial agents) with CAMHB medium. Plates was incubated at 35 °C for 24 h, and the lowest concentration of the agent that inhibits the growth of *S. haemolyticus* considered the MIC. MBC value was determined by adding 50 µL drug-containing bacterial solution (drug concentration ≥ MIC) on the surface of agar, and the lowest concentration without colony formation was MBC. Experiments were conducted in triplicate.

*S. aureus* (ATCC@29,213) was taken as the quality control strain and cefazolin was applied as the quality control drug. The assay was considered valid and reliable if the MIC of the QC strain was within the range of 0.25 to 1 µg/mL under the conditions of parallel operation.

### Effects of flavaspidic acid BB on biofilm

Antibacterial susceptibility tests were performed on biofilms at distinct developmental phases (initial adhesion, proliferation, and maturation). Briefly, each strain was inoculated on the surface of the NA medium and incubated at 35 °C for 24 h. A 200-µL aliquot of the inoculum was inoculated onto a 96-well plate at 35 °C for 0, 2, 4, 8, 12, 16, 24, 36, 48, and 72 h. Next, 100 µL of tryptic soy broth (TSB) medium and 10 µL of CCK-8 reagent were added to each well, and the culture medium was discarded. After incubation at 37 °C for 1 h without agitation, bacterial growth (OD450) was determined using a microplate reader (BIO-RAD, USA). Biofilm assays were performed to investigate antibacterial agents against the isolate [32]. Microtiter plates were filled with 200 µL of 10^6^ CFU/mL in TSB medium and incubated at 37 °C for 4, 8, and 24 h without shaking. To establish biofilms, planktonic bacteria were discarded daily and replaced with fresh TSB containing ½MIC, 1MIC, and 2MIC of flavaspidic acid BB and MUP. Simultaneously, a blank control group was established. After incubation at 35 °C for 24 h, 20 µL of CCK-8 reagent was added to each well. After incubation for 1 h, the absorbance was determined at 450 nm using a microplate reader. (In this study, clinically sensitive strain SHA 3 was selected as the experimental strain, which was the same in subsequent experiments.).

### Scanning electron microscopy (SEM) of bacteria samples

Sterile round slides (14 mm) were placed in a 24-well plate with tweezers, 1 mL of the inoculum (10^6^ CFU/mL) was added to each well, incubated at 35 °C for 4, 8 and 24 h, and the supernatant was aspirated. The liquid and suspension bacteria were washed three times with sterile phosphate-buffered saline (PBS) to remove the planktonic bacteria on the surface, and 1 mL of fresh TSB containing ½MIC, 1MIC, and 2MIC of flavaspidic acid BB and MUP was added; a growth control group (no drug contained) was also set at the same time. After incubation at 35 °C for 24 h, the medium was aspirated. The sterile round slides were washed three times with sterile PBS solution to obtain a biofilm of *S. haemolyticus* after intervention with different drug concentrations. The specimens were fixed in 2.5% glutaraldehyde for 24 h, submerged in ethanol solutions at concentrations of 30%, 50%, 70%, 80%, and 95% twice for 15 min, and then submerged in 100% ethanol for 1 h. After 24 h of desiccation at 37 °C, they were mounted on aluminum stubs with copper tape. After dehydration, ethanol was removed and replaced with 100% tert-butanol for 30 min. Then, they were coated with gold in a low-pressure atmosphere using an ion sputter coater [24, 33]. The surface topography of the bacterial cells was visualized and photographed using SEM (JSM-7610FPlus, JEOL, Japan).

### Molecular docking

Molecular docking was performed using SYBYL. The protein structures of eukaryotic initiation factor 2 α (eIF2α) (PDB ID: 1Q46), NADH dehydrogenase Complex I (PDB ID: 6G72), and ribonuclease P (RNase P) (PDB ID: 6D1R) were obtained from the RCSB protein database. Using the homologous modeling method, adenosine triphosphate (ATP) synthase and 70-kDa heat shock proteins (Hsp70) were obtained from the NCBI, and the three-dimensional structure of the corresponding template proteins was modeled by Swiss-Model. Details can be found in the supplementary materials. Briefly, the ligands were docked into the binding site of the corresponding protein in compliance with the protocol. The core module of molecular docking in SYBYL is Surflex-Dock. Surflex-Dock uses protomol to represent protein-binding pockets. The prototypical is a hypothetical molecule that perfectly matches the shape and properties of the protein active sites. If there are small ligand molecules in the protein, the active pocket can be determined based on this small molecule. If there is no known ligand small molecule, the location of the functional pocket can be roughly determined according to the key residues or automatically searched by software.

### Molecular Dynamics (MD) simulations

MD simulations were performed based on molecular docking using GROMACS 5.0. All MD simulations were performed using the GROMOS53a6 force field. The program selected the SPC/E water model and added Na^+^ or Cl^−^ to maintain electrical neutrality. The system was then heated from 0 to 300 K in the NVT ensemble of 100 ps and NPT ensemble of 100 ps with a small force constant on the enzyme to restrict any drastic changes. Finally, periodic boundary conditions of 30 ns were performed for the entire system at a normal temperature of 300 K in the production step.

### Effect of flavaspidic acid BB on Hsp70 and RNase P synthase

The concentration of bacterial suspension was diluted from 1.0 × 10^6^ CFU/mL to 1 × 10^5^ CFU/mL with TSB medium. The bacterial suspensions were added to a sterile centrifuge tube, followed by the addition of the drug as follows: blank control group, positive control group (½MIC, 1MIC, and 2MIC), and flavaspidic acid BB groups (½MIC, 1MIC, and 2MIC). The strains of each group were cultured at 35 ℃ for 24 h and then centrifuged (1000 rpm, 10 min). The isolated bacteria were re-suspended with cold PBS and ultrasound was performed 70 times under the ultrasonic cell powder machine. After the cells were broken, they were centrifuged at low temperature (4℃, 5000 rpm, 30 min). The supernatants after sub-packing were stored at -20℃ until analysis.

With MUP as the positive control, the effects of flavaspidic acid BB on Hsp70 and RNase P were investigated by HSP 70 ELISA kit (Jinmei, JM-1,201,601, China) and RNase P ELISA kit (Jinmei, JM-1,201,201, China) [34], respectively. The assays were carried out according to the manufacturer’s instructions.

### Statistical analysis

All assays were performed in triplicate and the values were expressed as mean ± SD. Analyses were performed using GraphPad Prism software version 8.0 (GraphPad Software, Inc., La Jolla, CA). The differences were evaluated with a one-way ANOVA. The differences were considered significant when p < 0.05.

## Results

### Antibacterial activities on *Staphylococcus haemolyticus*

The antibacterial results of all the compounds are shown in Table 1. The MIC values of flavaspidic acid BB against clinical strains of *S. haemolyticus* 1–16 was ranged from 5 µg/mL to 480 µg/mL. When the MIC value of E was ≥ 8 µg/mL, the strain was evaluated as a drug-resistant strain. When the MIC value of MUP was > 512 µg/mL, the strain was considered a high-level mupirocin-resistant strain. The MIC values of flavaspidic acid BB for SHA 3 and SHA 13 were 20 µg/mL and 30 µg/mL, respectively, and the MBCs were 40 µg/mL and 240 µg/mL, respectively. The MIC values of MUP, FD, and E against SHA3 and SHA13 were > 2560 µg/mL.

Meanwhile, the MIC of quality control strain *S. aureus* to cefazolin was 0.71 µg/mL, which was within the range of 0.25-1 µg/mL, indicating that the results of this experiment were reliable.

**Table 1:** MICs and MBCs of flavaspidic acid BB against 16 clinical strains of *Staphylococcus haemolyticus*

Isolates	BB(µg/mL)		MUP(µg/mL)		E(µg/mL)		FD(µg/mL)	
	MIC	MBC	MIC	MBC	MIC	MBC	MIC	MBC
SHA 1	33.3	133.3	240	>2560	>2560	>2560	>2560	>2560
SHA 2	30	53.3	<5	<5	>2560	>2560	>2560	>2560
SHA 3	20	40	2560	>2560	>2560	>2560	>2560	>2560
SHA 4	26.6	66.6	2560	>2560	266.6	1280	>2560	>2560
SHA 5	26.6	53.3	<5	<5	>2560	>2560	>2560	>2560
SHA 6	26.6	106.6	<5	<5	480	1280	>2560	>2560
SHA 7	13.3	320	13.3	320	<5	<5	>2560	>2560
SHA 8	10	80	10	120	<5	<5	>2560	>2560
SHA 9	<5	<5	<5	30	<5	<5	2560	>2560
SHA 10	30	90	<5	<5	1280	>2560	>2560	>2560
SHA 11	15	120	<5	<5	<5	<5	2560	>2560
SHA 12	480	640	1280	>2560	<5	<5	>2560	>2560
SHA 13	30	240	2560	>2560	2560	>2560	>2560	>2560
SHA 14	15	200	240	320	480	640	2560	>2560
SHA 15	20	80	<5	<5	2560	>2560	>2560	>2560
SHA 16	80	80	<5	<5	<5	<5	1280	1280

SHA 1 ~ 16 means clinical strains of *S. haemolyticus* 1 ~ 16; For all data n = 6

### Effect of flavaspidic acid BB on *Staphylococcus haemolyticus* Biofilm

**Fig. 1:**
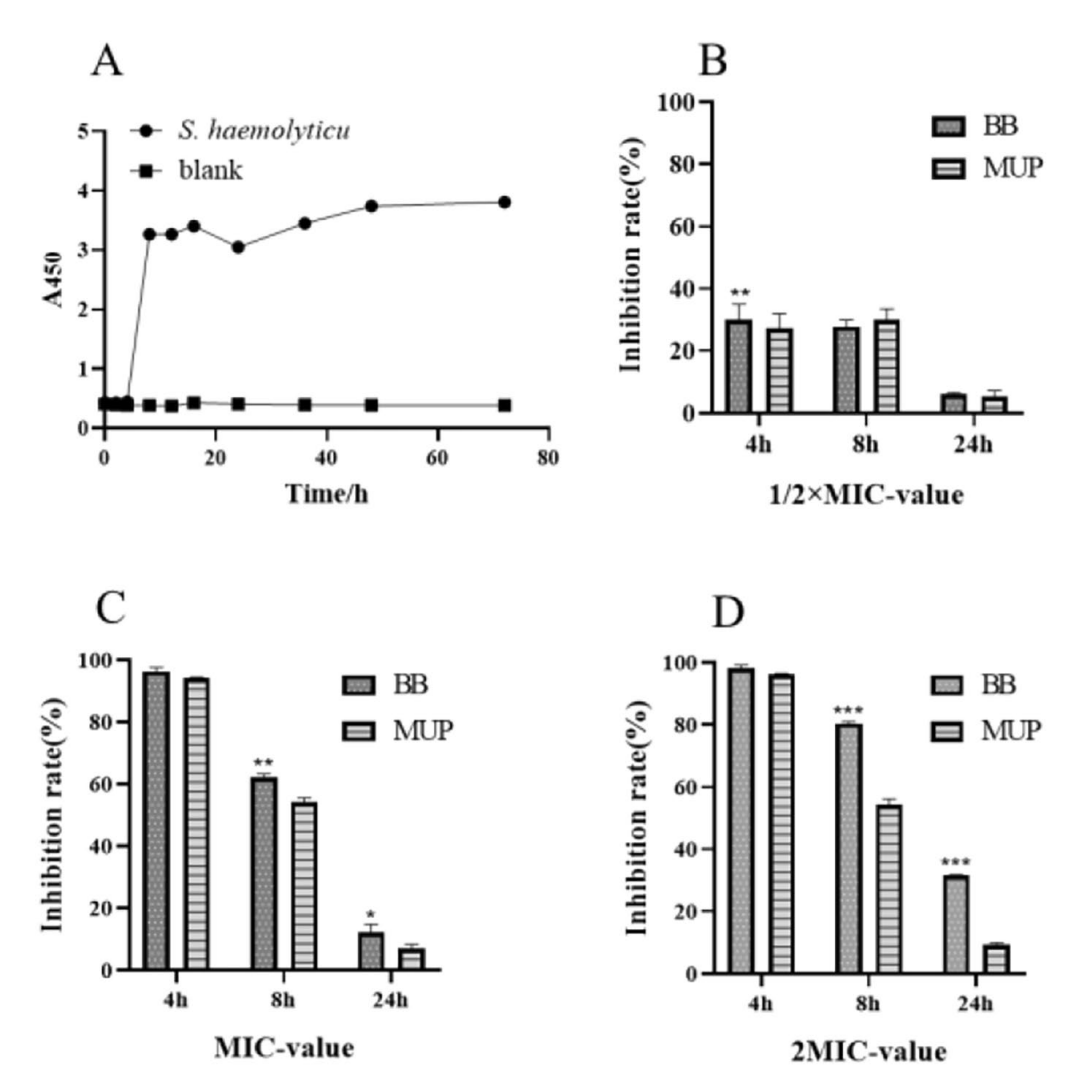
The effects of different drugs on the biofilm of *S. haemolyticus* at different growth stages. (**A**) Biofilm growth curve of *S. haemolyticus*. (**B**) Inhibitoin effect of ½MIC-value (10 µg/mL) Flavaspidic acid BB and Mupirocin (1280 µg/mL) on biofilm. (**C**) Inhibitoin effect of 1MIC-value (20 µg/mL) Flavaspidic acid BB and Mupirocin (2560 µg/mL) on biofilm. (**D**) Inhibitoin effect of 2MIC-value (40 µg/mL) Flavaspidic acid BB and Mupirocin (5120 µg/mL) on biofilm. *P < 0.05, **P < 0.01, *** P < 0.001 when compared to Mupirocin group

Previous experimental studies have shown that flavaspidic acid BB has a significant inhibitory effect on *S. haemolyticus*. The dynamic process of *S. haemolyticus* biofilm formation is shown in Fig. 1(A). The strain entered the initial adhesion stage when cultured at 35 °C for 4 h, formed microcolonies at 4–8 h, formed biofilm after 8 h, and completed the mature biofilm at 24 h. Therefore, the effect of flavaspidic acid BB on biofilm was further explored to provide a basis for the treatment of bacterial biofilm-related infection. The inhibitory effect of flavaspidic acid BB on the biofilm at different growth stages was determined by the CCK-8 assay (Fig. 1(B-D)). When cultured for 4 h to the initial adhesion stage, the results showed that inhibition rates (IR) of biofilm formation in the 1MIC (20 µg/mL) and 2MIC (40 µg/mL) groups were 96.39% and 98.16%, respectively, showing that it had a semblable inhibition effect with the positive drug MUP at 1MIC (2560 µg/mL), and 2MIC (5120 µg/mL). After incubation for 8 h to reach the proliferation stage, the IR of flavaspidic acid BB on biofilm formation at ½MIC, 1MIC, and 2MIC were 27.79%, 62.02%, and 80.48%, respectively. The inhibitory effects of MUP at ½MIC (1280 µg/mL), 1MIC (2560 µg/mL), and 2MIC (5120 µg/mL) at the same time were only 29.98%, 54.11%, and 54.38%, respectively. Compared with the 1MIC and 2MIC MUP groups, the 1MIC and 2MIC flavaspidic acid BB group had a better inhibitory effect (P < 0.01 and P < 0.001, respectively). Finally, after incubation for 24 h, the IR of flavaspidic acid BB on biofilm formation at ½ MIC, 1MIC, and 2MIC were 6.17%, 12.25%, and 31.63%, respectively. However, the IR of MUP was only 5.6-9.26%. Compared with the 2MIC MUP group, the 2MIC flavaspidic acid BB group had a better inhibitory effect (P < 0.001). These results showed that flavaspidic acid BB inhibited biofilm formation in a concentration-dependent manner, and that its ability to inhibit biofilm formation was better than that of MUP.

### Investigation of *S. heamolyticus* biofilm by SEM

**Fig. 2:**
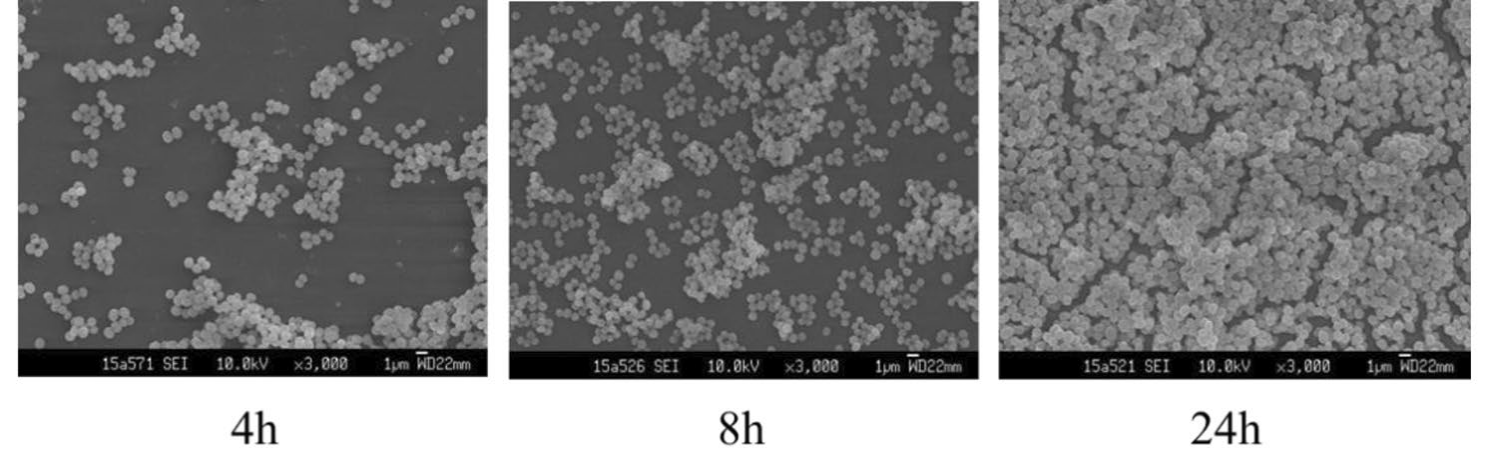
The characteristics of *S. heamolyticus* strain biofilms at different timeline (4 h, 8 h, 24 h) from SEM. Magnification: ×3,000

**Fig. 3:**
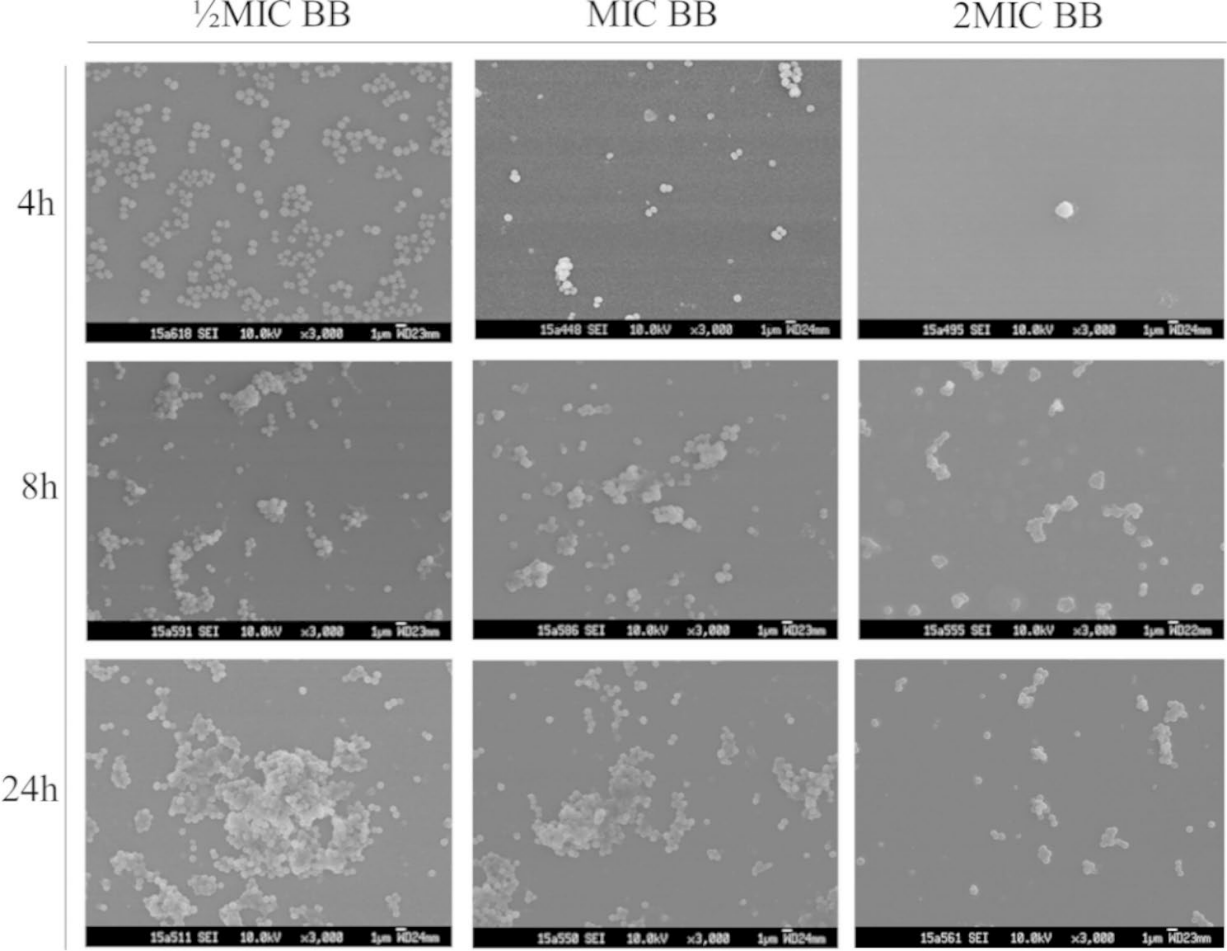
The characteristics of *S. heamolyticus* strain biofilms at different timeline (4 h, 8 h, 24 h) under flavaspidic acid BB from SEM. Magnification: ×3,000

**Fig. 4:**
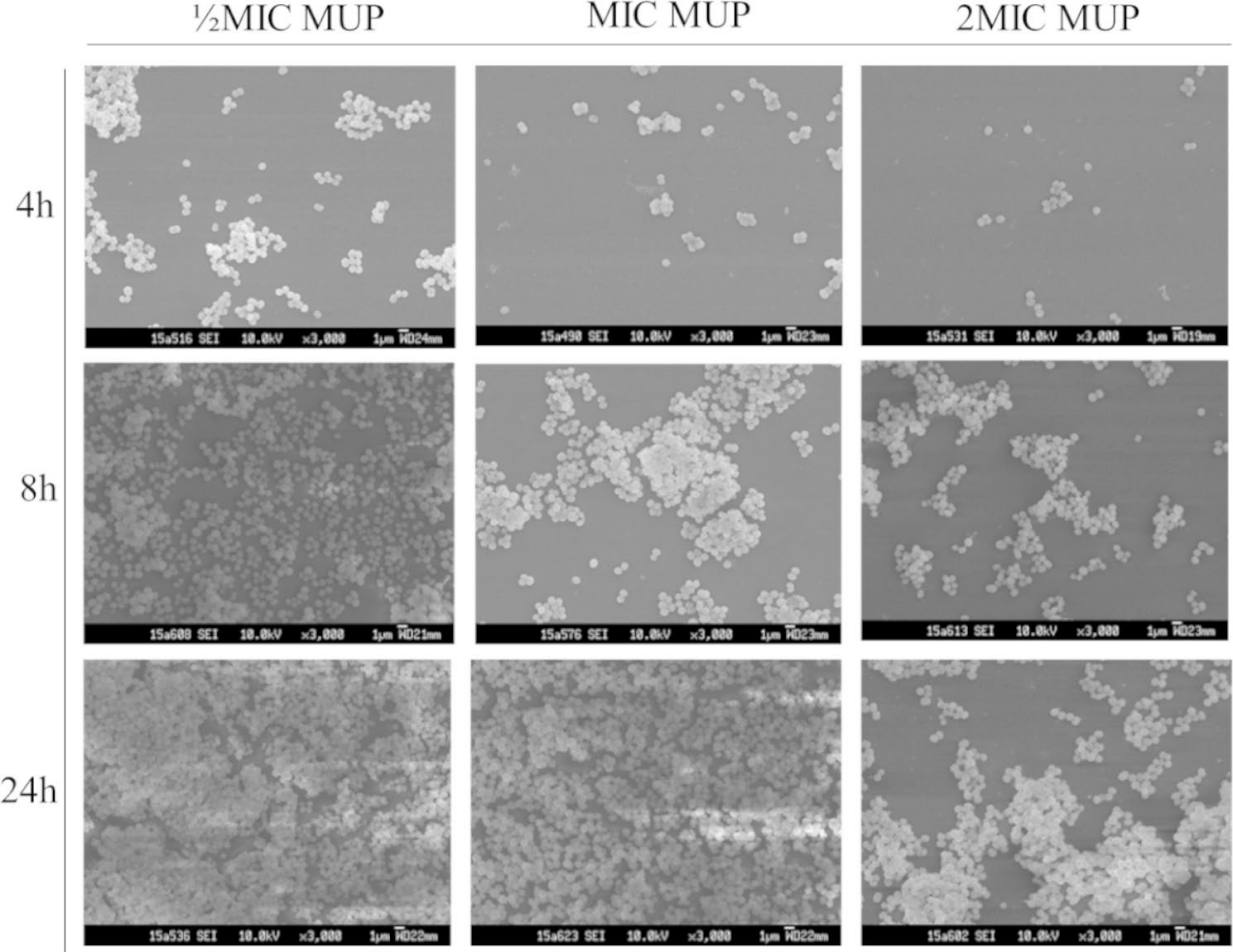
The characteristics of *S. heamolyticus* strain biofilms at different timeline (4 h, 8 h, 24 h) under MUP from SEM. Magnification: ×3,000

SEM was used to observe the morphological changes in the biofilms of *S. haemolyticus* after incubation for 4, 8, and 24 h (Fig. 2) and administration of different concentrations of flavaspidic acid BB and MUP (Figs. 3 and 4). SEM images showed that the control group of *S. haemolyticus* biofilm had the typical architecture of a mature biofilm (Fig. 2). In the sample group (flavaspidic acid BB) and the positive drug group (MUP), the bacterial community formed by the biofilm reduced with an increase in drug concentration at the same time. For instance, after 4 h of incubation, only sparse colonies were formed at a concentration of 10 µg/mL (½MIC) of flavaspidic acid BB, and no complete biofilm was established. At a concentration of 20 µg/mL (1MIC) and 40 µg/mL (2MIC), the cell volume was reduced and no biofilm was formed, even when single bacterial strains were distributed. The colonies at ½MIC in the MUP group was sparse and divided. The biofilm at 1MIC and 2MIC in the MUP group was evenly distributed. After 8 h of incubation, adherence occurred at a concentration of 10 µg/mL (½MIC) and 20 µg/mL (1MIC) in the flavaspidic acid BB groups, forming colonies of different sizes. The structure of the biofilm was sparse and did not form a complete biofilm morphology. The colonies at 2MIC in the flavaspidic acid BB group comprised single uniformly distributed cells with no adhesion between cells and no obvious biofilm structure. Dried bacteria formed by cell swelling, rupture, and exudation of cell volume were also observed. The colonies of the ½MIC MUP group were uniformly distributed, with an obvious biofilm structure. Colonies of the 1MIC and 2MIC MUP groups were unevenly distributed and adhered to each other, but no systematic biofilm morphology was observed. After incubation for 24 h, sparse biofilm aggregates were formed between the bacteria in the ½MIC flavaspidic acid BB group. In the 1MIC and 2MIC flavaspidic acid BB groups, the adhesion between bacteria was reduced and the biofilm structure tended to collapse. The biofilm morphology of the ½MIC and 1MIC MUP groups was similar to that of the normal growth group, but there was no inhibitory effect on biofilm formation. Colony formation in the 2MIC MUP group was sparser than that in the growth group, but the structure of the biofilm could be clearly seen.

### Docking studies

**Fig. 5:**
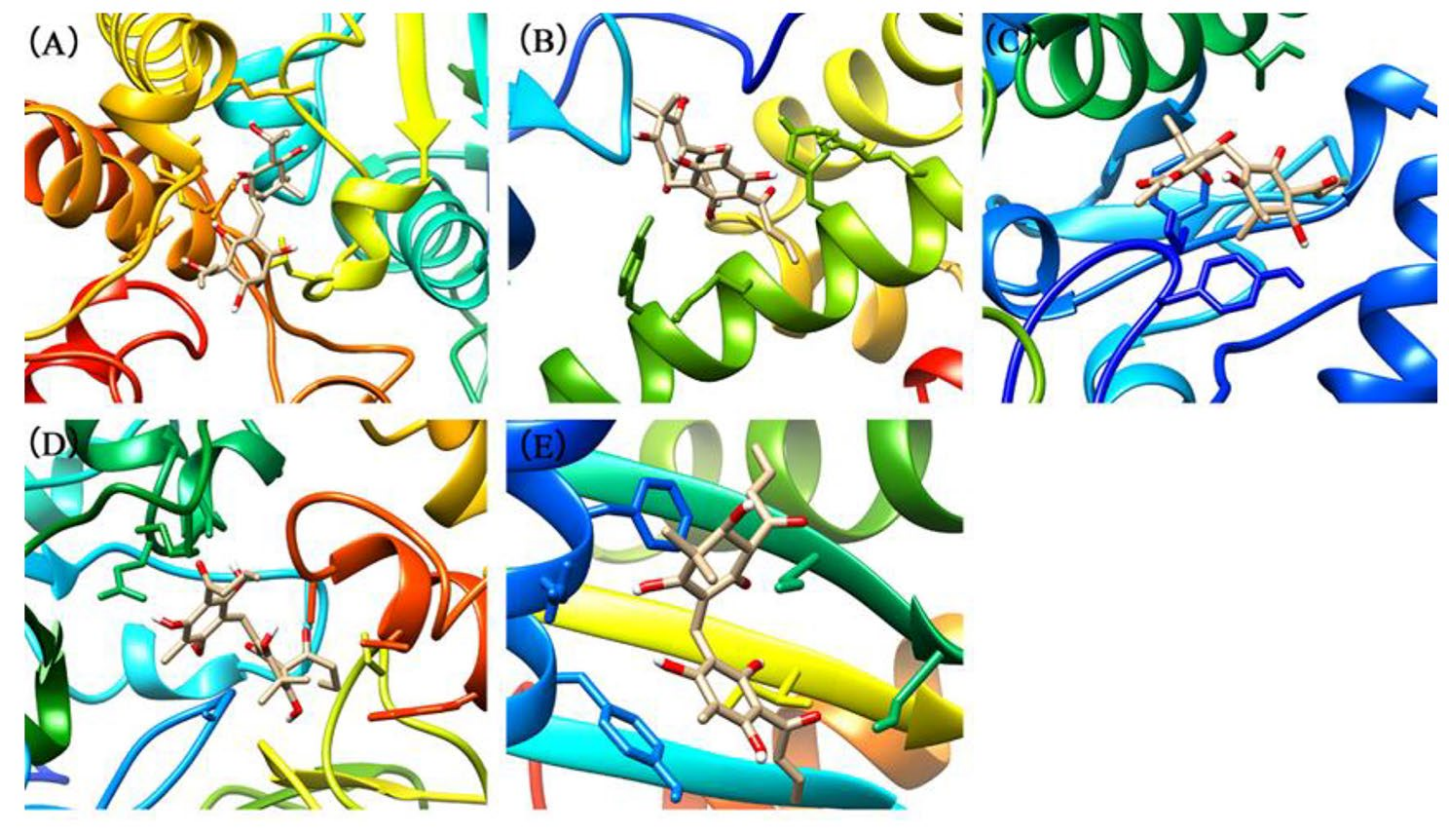
3D docking conformation of flavaspidic acid BB with five target enzymes: (**A**) 3D docking conformation of flavaspidic acid BB with ATP synthase; (**B**) 3D docking conformation of flavaspidic acid BB with eIF2α protein; (**C**) 3D docking conformation of flavaspidic acid BB with Hsp70 protein; (**D**) 3D docking conformation of flavaspidic acid BB with NADH dehydrogenase complex I; (**E**) 3D docking conformation of flavaspidic acid BB with RNase P protein

**Fig. 6:**
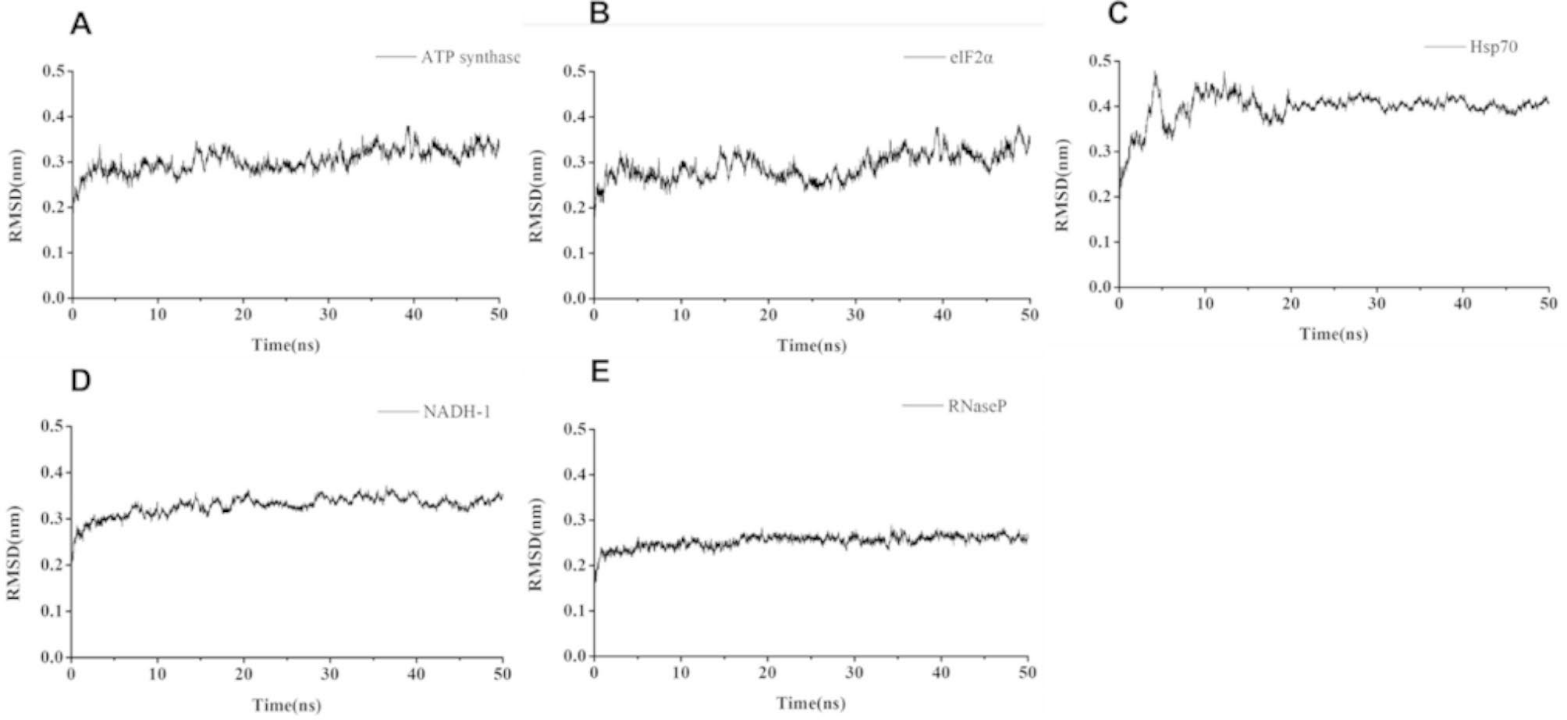
Molecular dynamics simulation of flavaspidic acid BB with ATP synthase, eIF2α, Hsp70, NADH-1, RNase P: (**A**) molecular dynamics simulation of flavaspidic acid BB with ATP synthase; (**B**) molecular dynamics simulation of flavaspidic acid BB with eIF2α;(**C**) molecular dynamics simulation of flavaspidic acid BB with Hsp70;(**D**) molecular dynamics simulation of flavaspidic acid BB with NADH-1;(**E**) molecular dynamics simulation of flavaspidic acid BB with RNaseP.

The binding energies of the complexes between flavaspidic acid BB and the active sites of the receptor are shown in Table 2. The binding energy of RNase P and Hsp70 to flavaspidic acid BB was lower, indicating that the binding of these two target enzymes to flavaspidic acid BB was more stable. The docking conformations of flavaspidic acid BB with the active sites of eIF2α, Hsp70, RNase P, ATP synthase, and NADH dehydrogenase Complex I are shown in Fig. 5. Docking results showed that the binding modes of flavaspidic acid BB to protein sites were similar, which interacted through hydrogen bonding formed by hydrogen bonds. Briefly, flavaspidic acid BB interacted with key amino acids LEU342, TYR381, LYS382, GLN385, ALA389, ILE390, LEU391, ARG408, and GLN411 in ATP synthase. When flavaspidic acid BB interacted with the eIF2 α protein, we defined amino acids in the 5 Å range near flavaspidic acid BB as their key amino acid. The key amino acids were LEU36, TYR101, SER104, LYS105, HIS108, and ARG112. Furthermore, flavaspidic acid BB interacted with Hsp70 protein by binding to THR14, TYR15, TYR41, VAL59, PHE68, THR265, GLU268, ARG269, ARG272, NADH dehydrogenase, and mitochondrial complex I could combine flavaspidic acid BB with GLY70, ALA71, GLU101, GLU188, GLU189, LYS206, SER303, THR304, ILE404, CYS405, ALA406, ASP409, GLY410, and TRP413. Finally, flavaspidic acid BB could combine RNase P protein with ASN12, PHE15, GLN16, TYR19, ILE47, SER48, SER50, and ILE85. To explore the interaction of compounds with proteins, we performed MD. The results of MD showed that the root-mean-square deviation values were at equilibrium conditions within 50 ns (Fig. 6). Additional information on molecular simulations can be found in the supplementary materials.

**Table 2:** The binding ability of flavaspidic acid BB to related proteins based on Molecular Dynamics

protein	Binding energy(kJ/mol)
RNase P	-168.729 ± 29.477
Hsp70	-139.495 ± 21.844
eIF2 α	-116.936 ± 17.017
ATP synthase	-96.712 ± 11.436
NADH dehydrogenae Complex I	-73.861 ± 31.428

### Effect of flavaspidic acid BB on Hsp70 and RNase P synthase

**Fig. 7:**
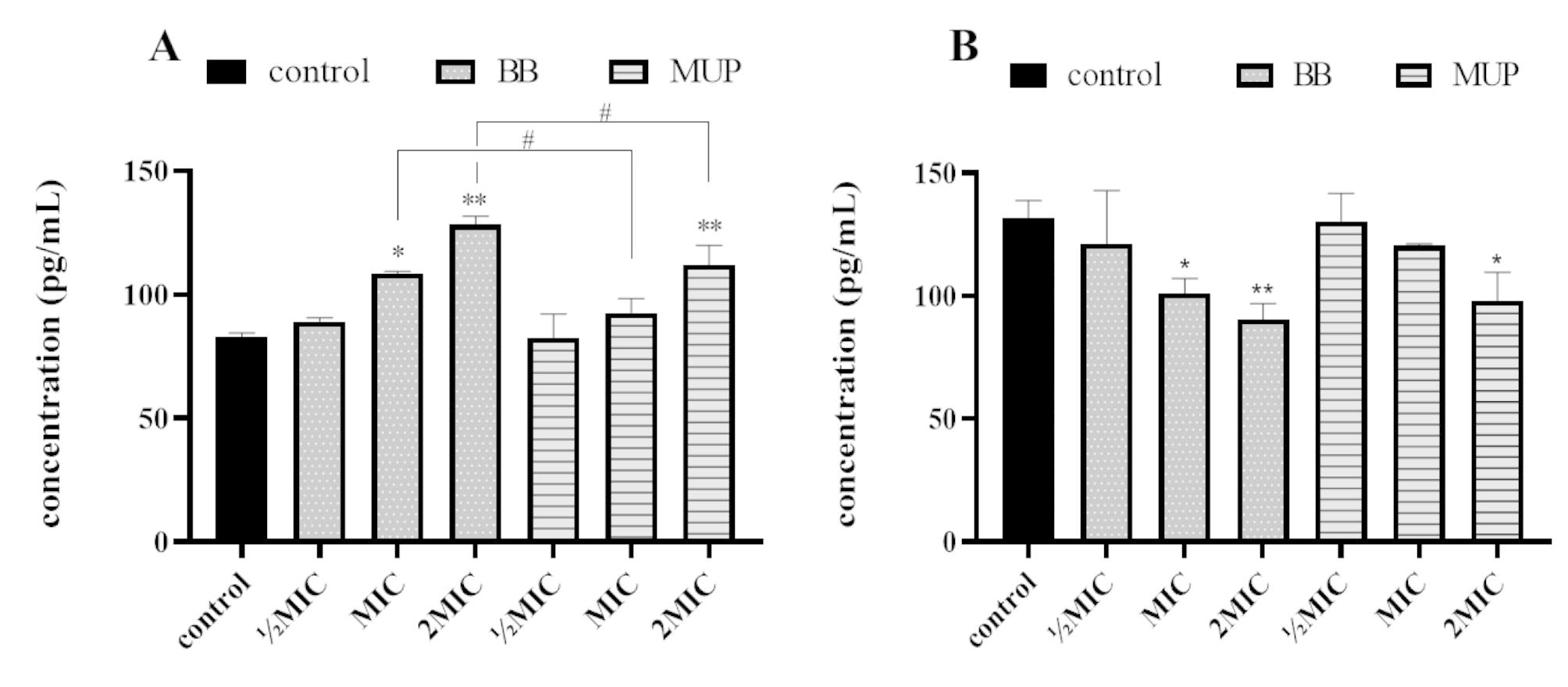
The effects of different drugs on expression of Hsp70 (**A**) and RNase P (**B**). *P < 0.05, **P < 0.01 when compared to control group, ^#^P < 0.05 when compared to MUP group

According to the results of molecular docking and molecular dynamics simulation, two target enzymes, Hsp70 and RNase P, were selected for the next verification test to explore the antibacterial mechanism of flavaspidic acid BB. The effects of flavaspidic acid BB and MUP on the expression of Hsp70 and RNase P are shown in Fig. 7. The results showed that the expression of Hsp70 increased in all concentration groups of flavaspidic acid BB and MUP compared to that in the growth control group. The expression of Hsp70 in different concentration groups of flavaspidic acid BB increased, which indicated that flavaspidic acid BB caused bacteria to enter into the stress reaction, inhibited protein degradation, and hindered protein utilization and re-synthesis. Further, with the increase in flavaspidic acid BB concentration, the expression of Hsp70 increased, especially in the 1MIC and 2MIC groups (P < 0.05 and P < 0.01, respectively). The expression of Hsp70 was not adversely affected by the concentration of MUP at ½MIC. The expression of Hsp70 was increased in the concentration of MUP in the 1MIC group and the expression of Hsp70 was significantly increased in the 2MIC group (P < 0.01). Flavaspidic acid BB had a slightly stronger effect than MUP in the 1MIC and 2MIC groups (P < 0.05). The effects of flavaspidic acid BB and MUP on the expression of RNase P showed that compared to the expression of RNase P in the growth control group, that in all concentration groups of flavaspidic acid BB (½MIC, 1MIC, 2MIC) decreased. Remarkably, flavaspidic acid BB in the significantly inhibited RNase P in the 1MIC and 2MIC groups (P < 0.05 and P < 0.01, respectively). MUP had a significant inhibitory effect on RNase P in the 2MIC group (P < 0.05). The inhibition of flavaspidic acid BB was slightly better than that of MUP. With an increase in the concentration of BB, the inhibition increased, indicating that flavaspidic acid BB had a concentration-dependent effect on the expression of RNase P.

## Discussion

The increase in drug-resistant opportunistic pathogenic bacteria, especially of antibiotic-resistant *S. haemolyticus*, has led to difficulties in the treatment of SSTI. Many studies have shown that biofilm is an important factor that affects bacterial resistance [35–37], which has also caused serious clinical problems. Therefore, the development of effective, safe, and low-resistance anti-biofilm drugs is urgently required.

In our study, we used the CLSI M07-A9 method for the determination of the MICs and MBCs of flavaspidic acid BB and antibiotics against 16 clinical strains of *S. haemolyticus*. E, MUP, and FD were used as positive controls to evaluate their activities. There were two clinical isolates (SHA 3 and SHA 13) in which the MIC value for the antibiotics reached 2560 µg/mL. These results indicated that the susceptibility of these isolates to E, MUP, and FD presented a wide range and were dependent on the strain. Some isolates of *S. haemolyticus* showed primary resistance to MUP.

Growth within the biofilm increases the chance of *Staphylococci* protecting themselves from host defenses, antibiotic treatments, and biocides [10]. There is much evidence [38, 39] indicating that one of the significant factors associated with drug resistance is the presence of biofilm, which causes serious clinical problems. Therefore, how to effectively prevent the formation of biofilms is still a challenge. Biofilm formation can be described as a dynamic process involving successive stages. Commonly, it is described as four main stages—attachment, proliferation, maturation, and detachment (dispersal). In our study, the strains of *S. haemolyticus* were sensitive to anti-bacterial agents during the course of biofilm formation and less sensitive after mature biofilm formation. We focus on flavaspidic acid BB and MUP, which can effectively inhibit the biofilm formation of *S. haemolyticus* in the initial adhesion stage. This was in agreement with the SEM findings of our study.

SEM permits the visualization of the detailed surface morphologies of microbial biofilms and their structures. The results demonstrated that *S. haemolyticus* formed biofilms on polystyrene surfaces. In our study, the morphological changes in the *S. haemolyticus* biofilm at 4, 8, and 24 h of cultivation with different concentrations of flavaspidic acid BB and MUP were evaluated. When treated with different concentrations of flavaspidic acid BB, the biofilm morphology showed a distinctly different appearance. Compared to the control group, 1MIC (20 µg/mL) and 2MIC (40 µg/mL) groups had a certain inhibitory effect on the biofilm in the initial adhesion stage. The result was similar to that of the positive control (1MIC and 2MIC), while the inhibition of biofilm formation by flavaspidic acid was slightly superior to that of the MUP. However, as the biofilm of the strain matured after 24 h of cultivation, the anti-biofilm ability of the BB gradually weakened, which is due to the integrated biofilm system that effectively prevents toxins such as antibiotics and disinfectants from reaching their targets [40, 41].

In addition, to accurately and simply identify the target quickly, we used molecular docking and MD simulations to predict potential targets and conducted enzyme dynamic experiments for verification. NADH dehydrogenase Complex I and ATP synthase are key enzymes in the respiratory chain and are abundant in mitochondrial DNA. eIF2α and Hsp70 proteins can mediate the genes related to the stress response of the cytoplasmic reticulum and cells. Hsp70 is a pan-ligase complex in the endoplasmic reticulum-related degradation pathway. It can inhibit protein degradation and hinder the utilization and re-synthesis of proteins [42]. RNase P participates in the structural modification and processing of tRNA precursors [43]. These enzymes are closely related to DNA synthesis and protein synthesis and, to a certain extent, affect the structure of the biofilm. By quantifying the binding affinity of flavaspidic acid BB with five target enzymes by binding free energy, it can be concluded that among the five target enzymes, Hsp70 and RNase P receptors bind more stably to flavaspidic acid BB. ELISA showed that the expression of Hsp70 in different concentration groups of BB increased. In prokaryotes, Hsp70 mutations cause abnormally high expression of Hsp70, indicating that Hsp70 acts as a negative regulator of Hsp70 expression, inhibits protein degradation, and hinders protein utilization and re-synthesis [44]. Moreover, compared to the expression of RNase P in the growth control group, that in all concentration groups of flavaspidic acid BB decreased. There is also evidence that depletion of RNase P affects bacterial growth, possibly due to a lack of functional tRNA for translation [45]. It is suggested that the antibacterial mechanism of flavaspidic acid BB may be achieved by inhibiting the utilization and synthesis of proteins and the synthesis of tRNA, thus inhibiting bacterial growth and biofilm formation to a certain extent.

In summary, the results showed that flavaspidic acid BB significantly inhibited the planktonic bacteria and biofilm of clinical strains of *S. haemolyticus*. The change in the inhibitory effect of flavaspidic acid BB on the biofilm indicated that the inhibition of flavaspidic acid BB on the biofilm at different growth stages was distinct. Especially in the initial adhesion stage, the inhibitory effect of flavaspidic acid BB on the biofilm was stronger. Overall, the inhibitory effect of flavaspidic acid BB was slightly better than that of MUP, indicating that flavaspidic acid BB had a good inhibitory effect on biofilm formation. The ELISA assay showed that flavaspidic acid BB promoted the activity of Hsp70 and inhibited the activity of RNase P, revealing that flavaspidic acid BB could effectively inhibit the utilization and re-synthesis of protein and tRNA synthesis, thus inhibiting bacterial growth and biofilm formation to a certain extent. However, this study only established the static biofilm in vitro, while the biofilm formation in the biological organism varies greatly due to the complex environment and many influencing factors, and the in vivo model of biofilm should be studied subsequently. Further studies will focus on the determination of BB cytotoxicity due to safety concerns for clinical application.

## Conclusion

Our study focused on flavaspidic acid BB, which could inhibit the high levels of mupirocin-resistant strains of *S. haemolyticus* and its biofilm formation. Thus, it can be used as a new antimicrobial agent for resistant strains, which can provide a respite for global bacterial infection in SSTI by solving the problem of increasing the resistance rate of commonly used topical antibiotics in clinics.

### Electronic supplementary material

Below is the link to the electronic supplementary material.

**Additional file 1**: Supplementary information for prediction of flavaspidic acid BB action target tests based on molecular docking and molecular dynamic simulation

## Data Availability

All data generated or analysed during this study are included in this published article and its supplementary information files. The datasets used and analysed during the current study available from the corresponding author on reasonable request.

## Abbreviations

S. haemolyticus (SHA): Staphylococcus haemolyticus
SSTI: Skin and Soft Tissue Infections
MIC: Minimum inhibitory concentration
MBC: Minimum bactericidal concentration
MUP: Mupirocin
BB: Flavaspidic acid BB
E: Erythromycin
FD: Fusidic acid
NA: Nutrient Agar
CAMBH: Caton-adjusted Mueller-Hnton Broth
TSB: Tryptone soy broth
CFU: Colony-forming unit
CCK-8: Cell counting kit-8
SEM: Scanning electron microscopy
MD: Molecular dynamics
eIF2α: Eukaryotic initiation factor 2 α
RNase P: Ribonuclease P
ATP: Adenosine triphosphate
Hsp70: 70-kDa heat shock proteins
NPT: Isothermal-isobaric ensemble
NVT: Canonical ensemble
CLSI: Clinical and Laboratory Standards Institute
ATCC: American Type Culture Collection
