# Supplementary material for: Antibacterial activity and antibacterial mechanism of flavaspidic acid BB against Staphylococcus haemelyticus
Source: BMC Microbiol. 2023 Sep 29;23:276. doi: 10.1186/s12866-023-02997-5 (PMC10540430; doi:10.1186/s12866-023-02997-5)
Supplement: Supplementary file 1 — Additional file 1: Supplementary information for prediction of flavaspidic acid BB action target tests based on molecular docking and molecular dynamic simulation [file 12866_2023_2997_MOESM1_ESM.docx]

# Supplementary information for prediction of flavaspidic acid BB action target tests based on molecular docking and molecular dynamic simulation

## Ligand pretreatment

The structure of flavaspidic acid BB is shown in Fig. S1. Gauss-View 5.0 was used to construct the structure of flavaspidic acid BB, and gaussian 09 was used to optimize the structure under B3LYP/6-31 group.

**Fig. S1** The structure of flavaspidic acid BB (CAS: 114-42-1).

## The information of related protein in molecular simulation

Table S1 shows the information about five target enzymes used in molecular docking and molecular dynamics simulations.

**Table S1** Information of related proteins selected in Molecular Docking and Molecular Dynamics simulation

| protein | Full name | PDB ID |
| --- | --- | --- |
| ATP synthase | ATP synthase | Using 1COW as a template, we constructed the protein structure of mouse by Swiss-model using homology modeling method. |
| eIF2 α | Eukaryotic translation initiation factor 2 | 1Q46 |
| Hsp70 | Heat Shock Protein 70 chaperone Dnak | Using 5NRO as a template, we constructed the protein structure of mouse by Swiss-model using homology modeling method. |
| NADH | NADH dehydrogenase and mitochondria Complex I | 6G72. It is a super protein with about 45 subunits, and we selected some ligands to carry out research. |
| RNase P | Ribonuclease-P- Protein subunit (RnpA) | 6D1R |

## The result of Molecular Docking

Table S2 shows the results of molecular docking. We usually take the Total-Score value as the evaluation index. The Total-Score scoring function takes the hydrophobic interaction, polar interaction, enthalpy and flux interaction between the active component and the target protein as the evaluation criteria. The higher the Total score value is, the more stable the binding between the ligand molecule and the receptor protein is. It is generally believed that the total score higher than 7 indicates that the ligand molecule has high binding activity to the receptor protein, between 5 and 7 indicates that it has good binding activity, between 3 and 5 indicates that it has a certain binding activity, and if less than 3, it is considered to be weak. The results of molecular docking showed that Total Score (Hsp70) > 7, Hsp70 had higher binding ability to BB. And Total Score (RNase P) > 5, RNase P and BB had good binding ability.

| protein | Total Score | C Score |
| --- | --- | --- |
| Hsp70 | 8.30 | 4 |
| RNase P | 5.67 | 4 |
| ATP synthase | 4.96 | 4 |
| NADH | 4.93 | 2 |
| eIF2 α | 2.75 | 5 |

**Table S2** The docking information of 5 proteins with flavaspidic acid BB

## The result of Molecular Dynamics

In order to further quantify the binding affinity between flavaspidic acid BB and related receptor proteins, MM-PBSA was used to calculate the binding free energy between flavaspidic acid BB and different proteins (Table S3). The lower the binding free energy is, the more stable the binding complex is. The binding free energy is composed of several different energies such as van der Waal energy and electrostatic energy. The molecular dynamics results showed that the binding of Hsp70 and RNase P to flavaspidic acid BB was more stable.

**Table S3** The binding ability of flavaspidic acid BB to related proteins based on Molecular Dynamics

| protein | van der Waal energy(kJ/mol) | Electrostatic energy(kJ/mol) | Polar solvation energy(kJ/mol) | SASA energy(kJ/mol) | Binding energy(kJ/mol) |
| --- | --- | --- | --- | --- | --- |
| RNase P | -136.916±8.476 | -100.991±29.875 | 83.751±20.945 | -14.573±1.041 | -168.729±29.477 |
| Hsp70 | -158.349±17.218 | -75.756±31.439 | 112.4±24.768 | -17.591±1.470 | -139.495±21.844 |
| eIF2 α | -123.544±9.488 | -96.685±28.523 | 116.246±19.686 | -12.953±1.101 | -116.936±17.017 |
| ATP synthase | -152.728±10.757 | 1.512±6.540 | 41.882±9.887 | -14.378±1.128 | -96.712±11.436 |
| NADH | -100.001±10.002 | -0.274±0.047 | 25.768±41.322 | 0.098±3.041 | -73.861±31.428 |

## Summary of the results of molecular simulation

In summary, we investigated the stability and rationality of the binding of flavaspidic acid BB to five target enzymes by molecular docking and molecular dynamics simulation. Therefore, two target enzymes, Hsp70 and RNase P, were selected for the next verification test to explore the antibacterial mechanism of flavaspidic acid BB.
